# DC activated *via* dectin-1 convert Treg into IL-17 producers

**DOI:** 10.1002/eji.200838950

**Published:** 2008-12

**Authors:** Fabiola Osorio, Salomé LeibundGut-Landmann, Matthias Lochner, Katharina Lahl, Tim Sparwasser, Gérard Eberl, Caetano Reis e Sousa

**Affiliations:** 1 Immunobiology Laboratory, Cancer Research UK, London Research Institute, Lincoln's Inn Fields LaboratoriesLondon, UK; 2 Institut Pasteur, Laboratory of Lymphoid Tissue DevelopmentCNRS, URA 1961, Paris, France; 3 Institut für Medizinische Mikrobiologie, Immunologie & Hygiene, Technische Universität MünchenMunich, Germany

**Keywords:** DC, Dectin-1, Th17, Treg

## Abstract

Th cells producing IL-17 play a pro-inflammatory role at mucosal surfaces. Treg at the same sites dampen inflammation and prevent immunopathology. Th cells producing IL-17 (Th17) and Treg are thought to be distinct populations defined by expression of the transcription factors ROR-γt and Foxp3, respectively. Here, we show that mouse CD25^+^Foxp3^+^ Treg can be converted into a hybrid T-cell population characterized by the expression of Foxp3 and ROR-γt and the production of IL-17. Conversion was observed upon coculture with DC selectively activated *via* dectin-1, a C-type lectin receptor involved in fungal recognition, and depended on IL-23 produced by DC. Within the Foxp3^+^ population, only Foxp3^+^ROR-γt^+^ T cells but not Foxp3^+^ROR-γt^−^–T cells become Foxp3^+^IL-17^+^ T cells. These results indicate that some Foxp3^+^ T cells can produce IL-17 while retaining Foxp3 expression and suggest that Treg could play an unexpected pro-inflammatory role in some settings.

## Introduction

Our immune system identifies pathogens in part by recognizing signatures present in microbes, so-called PAMP, *via* specific germline encoded PRR [1]. Recognition of PAMP by PRR leads to an immediate innate response designed to contain infection and may also result in the mobilization of adaptive defense mechanisms. The translation of PAMP presence into adaptive immunity is carried out by specialized leukocytes called DC and results in distinct responses matched to the nature of the offending microbe. For CD4^+^ T cells, infection with intracellular bacteria tends to induce responses dominated by Th1 cells, whereas extracellular parasites often promote Th2-biased responses. Some bacteria and fungi promote a third type of response defined by the induction of Th cells producing IL-17 (Th17) [2–4]. Th17 cells are pro-inflammatory cells characterized by the expression of IL-17A, IL-17F, IL-21, IL-22, IL-23R and the transcription factors ROR-γt and ROR-α [5–7]. They have been implicated in several models of autoimmunity [8–11] but under normal conditions may play a role in protection from infection at mucosal surfaces [2]. IL-6 and TGF-β are key cytokines required for lineage commitment of murine Th17 [2, 8, 12] whereas IL-23 is a critical factor for sustaining these cells and is required for the acquisition of their pathogenic function *in vivo* [9, 13].

Treg are important regulators of potentially detrimental responses against normal self-constituents or commensal microbes and loss of Treg function leads to autoimmune and inflammatory diseases [14, 15]. Treg are characterized by the expression of CD25 and Foxp3, the latter being a transcription factor required for the maintenance of the Treg lineage [15, 16]. Like Th1, Th2 and Th17 cells, Foxp3^+^ Treg can be generated in the periphery from newly activated CD4^+^ T cells. The differentiation pathways of such inducible Treg and those of Th17 cells are closely related as both processes require the presence of TGF-β [8]. Notably, it has recently been found that fully differentiated Treg can themselves be converted into Th17 cells in mice [17–19] and humans [20]. This process is accompanied by extinction of Foxp3 expression and upregulation of ROR-γt [18]. Interestingly, some cells in the mouse can coexpress Foxp3 and ROR-γt [21, 22] and could represent an intermediate in this process or, alternatively, an intermediate cell in the differentiation of naïve T cells into Th17/Treg. Foxp3^+^ROR-γt^+^ cells behave as *bona fide* Treg, like their ROR-γt^−^ counterparts, and produce IL-10 but not IL-17 [22]. Thus, the available data suggest that, although Foxp3 and ROR-γt can be coexpressed in CD4^+^ T cells, Foxp3 expression and IL-17 production are mutually exclusive. This has led to the notion that regulatory and inflammatory T-cell programs are antagonistic [18, 21].

Dectin-1 is a Syk-coupled C-type lectin receptor for β-glucans involved in the innate recognition of fungi and some bacteria. Activation of the dectin-1 pathway in DC leads to the generation of Th17 cells both *in vitro* and *in vivo* [4]. Here, we show that dectin-1-activated DC can directly interact with CD25^+^Foxp3^+^ Treg and instruct them to become IL-17-producers. Notably, in contrast to previous studies, we find that such Treg do not extinguish the expression of Foxp3. Instead, they become a population of Foxp3^+^IL-17^+^ T cells that defies classification as either Treg or Th17 and could play an important role in inflammation.

## Results

### CD4^+^CD25^+^ T cells become IL-17 producers in response to curdlan-stimulated DC

**Figure 1:**
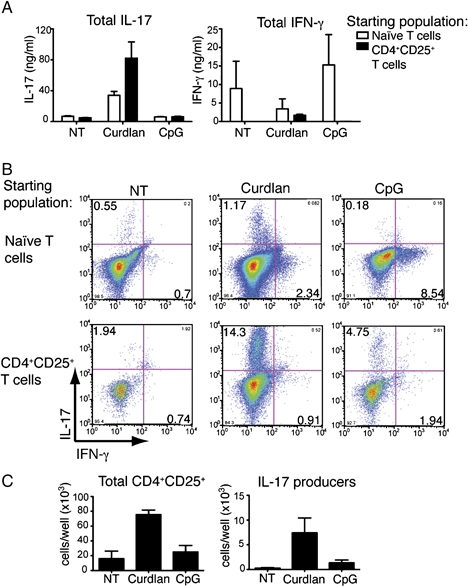
Curdlan-stimulated DC promote IL-17 production by CD4^+^CD25^+^ T cells. (A) FACS-sorted naïve T cells or CD4^+^CD25^+^ T cells from C57BL/6 mice were cocultured for 5 days with BMDC and soluble α-CD3 in medium alone or in the presence of curdlan or CpG. Half of the content of each well was restimulated on day 5 for 48 h with coated α-CD3 and cytokine production was determined by sandwich ELISA. (B) same as part A, but cells were restimulated on day 5 with PMA, ionomycin and brefeldin A for 4 h and the presence of intracellular cytokines was analyzed by flow cytometry. Data show IFN-γ and IL-17 after gating on CD4^+^ cells. (C) Total numbers of CD4^+^IL-17^+^ T cells or CD4^+^ T cells obtained on day 5 in cultures containing CD4^+^CD25^+^ T cells. Graph shows mean+SEM of three independent experiments. Data are representative of two to six independent experiments. N.D., not done.

We have previously reported the induction of Th17 cells in cocultures of CD4^+^ T cells and DC stimulated with the dectin-1 agonist, curdlan [4]. The emergence of a substantial fraction of IL-17-producing cells required the presence of both CD4^+^CD25^−^ and CD4^+^CD25^+^ cells [4]. To further investigate the role of CD4^+^CD25^+^ cells, we purified naïve (CD4^+^CD25^−^CD62L^hi^CD44^lo^) or CD4^+^CD25^+^ T cells from C57BL/6 mice and cultured each population individually with BM-derived DC (BMDC) plus α-CD3 mAb in the presence of curdlan or CpG (a TLR9 agonist). In cultures in which naïve T cells were the starting population, inclusion of CpG induced development of T cells that preferentially produced the Th1 cytokine IFN-γ rather than IL-17 upon restimulation (Fig. 1A and B). The inverse was observed in the presence of curdlan (Fig. 1A and B), as previously reported [4]. Notably, in CD4^+^CD25^+^ T-cell cultures, neither curdlan nor CpG led to the development of IFN-γ-producing cells but curdlan promoted the appearance of a large frequency and absolute number of cells able to produce IL-17 upon restimulation (Fig. 1A–C). This was accompanied by greater expansion of total CD4^+^CD25^+^ T cells in curdlan-containing cultures when compared with cultures containing CpG or lacking any innate stimulus (Fig. 1C), suggesting that factors produced by DC in response to curdlan favor proliferation and/or survival of CD4^+^CD25^+^ T cells and induce development of an IL-17-producing population.

### Curdlan-activated DC induce the generation of Foxp3^+^IL-17^+^ T cells

**Figure 2:**
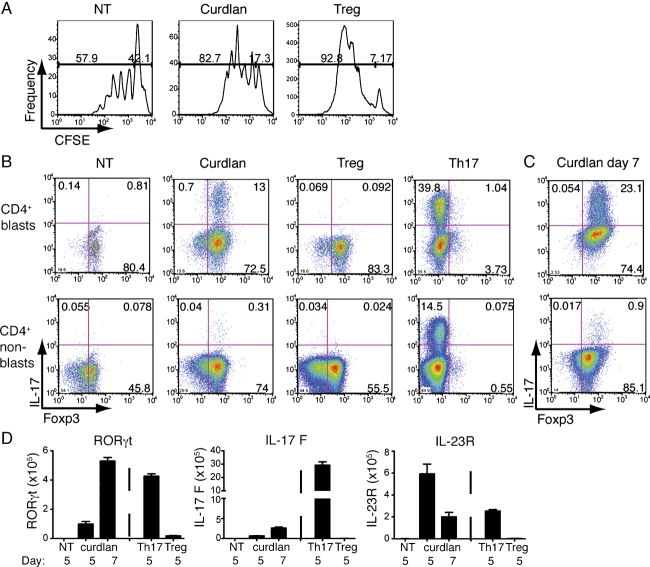
Curdlan-stimulated DC induce the generation of Foxp3^+^IL-17^+^ T cells. (A) CFSE-labeled CD4^+^CD25^+^GFP^+^ DEREG T cells were cultured for 5 days with wild-type BMDC and soluble α-CD3 in the absence (NT) or presence of curdlan or rhIL-2 (“Treg”), as indicated. Plots show CFSE profile after gating on CD4^+^ cells. (B) As in (A) but with the addition of a “Th17” control consisting of CD4^+^GFP^−^CD25^−^ T cells cultured with BMDC, α-CD3 and IL-6+TGF-β in the presence of neutralizing antibodies to IFN-γ and IL-4. All cells were restimulated on day 5 with PMA, ionomycin and brefeldin A for 4 h and the expression of Foxp3 and IL-17 was analyzed by gating separately on CD4^+^ cell blasts or non-blasts by flow cytometry. (C) Analysis of parallel cultures from part B on day 7. (D) RT-PCR analysis of transcripts for ROR-γt, IL-17F and IL-23R in the indicated cultures at 5 and 7 days. Data are representative of two to six independent experiments.

CD4^+^CD25^+^ T cells include activated T cells as well as a large population of Treg expressing Foxp3 [23]. We therefore set up cultures with a pure Foxp3^+^ T-cell population isolated by cell sorting from depletion of regulatary T-cell (DEREG) mice (or from radiation chimeras bearing BM from DEREG mice), which express GFP fused to the primate diphteria toxin receptor under the control of *foxp3* gene regulatory regions [24]. As for the total CD4^+^CD25^+^ T cells used previously, CD25^+^GFP^+^ DEREG T cells proliferated more extensively when curdlan was included in the cultures than when no innate stimulus was present (Fig. 2A). To account for a possible role of proliferation, we compared curdlan with IL-2 (Fig. 2A, Treg control), which acts as a Treg mitogen, and independently analyzed the blast and the resting T-cell population for expression of Foxp3 and the ability to produce IL-17 upon restimulation. Blast and non-blast cells were selected by forward- and side-scatter criteria, whereas Foxp3 expression was assessed by nuclear staining with a specific mAb to directly measure the presence of the protein at the time of analysis. As expected, we observed Foxp3^+^IL-17^−^ cells in Treg control cultures and Foxp3^−^IL-17^+^ cells in Th17 control cultures. However, in curdlan-containing cultures of Foxp3^+^ T cells and DC, we observed an accumulation of Foxp3^+^IL-17^+^ double-positive cells, which were especially noticeable in the blast fraction (Fig. 2B). T cells producing IL-17 and coexpressing Foxp3 continued to accumulate over time in curdlan-containing cultures and represented one-fourth of the cells by day 7 (Fig. 2C). At both time points, we detected the presence of transcripts associated with Th17 cell fate such as ROR-γt, IL23R and, to a lesser extent, IL-17F (Fig. 2D). Foxp3 transcripts were also found in these cultures, consistent with the staining data (data not shown). We conclude that, in response to BMDC stimulated with curdlan, Foxp3^+^ cells can give rise to a T cell capable of coexpressing Foxp3 and IL-17, which constitutes a distinct phenotype from classical Th17 or Treg.

### Foxp3^+^ROR-γt^+^ but not Foxp3^+^ROR-γt^−^ T cells become Foxp3^+^IL-17^+^ T cells

**Figure 3:**
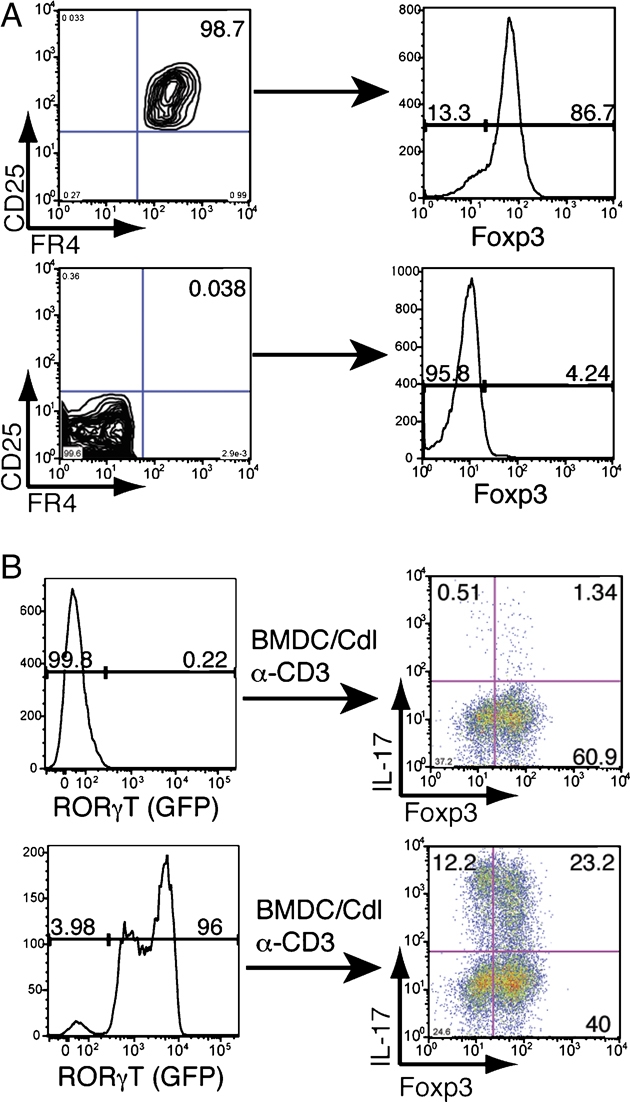
Foxp3^+^ROR-γt^+^ but not Foxp3^+^ROR-γt^−^ T cells become Foxp3^+^IL-17^+^ T cells in response to curdlan-activated BMDC. (A) FACS-sorted CD4^+^CD25^+^FR4^+^ and CD4^+^CD25^−^FR4^−^ T cells from C57BL/6 mice stained for intracellular Foxp3. (B) FACS-sorted CD4^+^CD25^+^FR4^+^GFP^+^ and CD4^+^CD25^+^FR4^+^GFP^−^ T cells from *Rorc*(*γt*)*-Gfp^TG^* chimeras were cocultured with wild-type BMDC and soluble α-CD3 in the presence of curdlan. Cells were restimulated on day 5 with PMA, ionomycin and brefeldin A for 4 h and the expression of Foxp3 and IL-17 on CD4^+^ cell blasts was analyzed by flow cytometry. Data are representative of two independent experiments.

Foxp3^+^ Treg are heterogeneous and include a fraction that coexpresses ROR-γt and produce little or no IL-17 [21, 22]. Given the importance of ROR-γt in differentiation of conventional Th17 CD4^+^ T cells [5], we wondered whether Foxp3^+^ROR-γt^+^ cells might represent the source of Foxp3^+^IL-17^+^ double-positive cells. We therefore compared ROR-γt^+^ and ROR-γt^−^ Treg isolated from chimeric mice reconstituted with BM from *Rorc*(*γt)-Gfp^TG^* mice, in which GFP reports ROR-γt expression [22]. To obtain Treg from those mice, we sorted cells based on the expression of CD4, CD25 and folate receptor 4, which closely mirrors Foxp3 expression [25]. We confirmed that the majority of sorted CD25^+^FR4^+^ CD4^+^ T cells express Foxp3 (Fig. 3A). We then prepared donor origin CD25^+^FR4^+^ T cells from *Rorc(γt)-Gfp^TG^* chimeric mice, which were sub-divided into GFP^+^ and GFP^−^ fractions and separately cultured with BMDC and α-CD3 in the presence of curdlan (Fig. 3B). Notably, only CD25^+^FR4^+^GFP^+^ and not CD25^+^FR4^+^GFP^−^ fractions were able to generate IL-17-producing cells (Fig. 3B). The majority of these cells coexpressed Foxp3 (Fig. 3B) as observed in experiments with DEREG T cells (see above). In addition, a proportion of IL-17^+^ cells were negative for Foxp3 (Fig. 3B), probably reflecting expansion of contaminating ROR-γt^+^Foxp3^−^ cells present in the starting population, which correspond to Th17 cells (Fig. 3A). We conclude that the Foxp3^+^ROR-γt^+^ but not the Foxp3^+^ROR-γt^−^ subset can be induced to produce IL-17 after culture with DC in the presence of a dectin-1 agonist.

### IL-23 drives the generation of Foxp3^+^IL-17^+^ T cells

**Figure 4:**
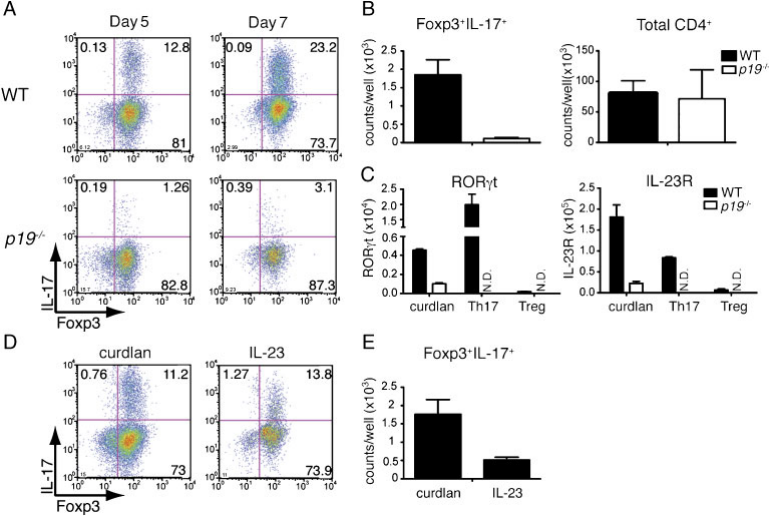
IL-23 drives the generation of Foxp3^+^IL-17^+^ T cells in response to curdlan-activated BMDC. (A) FACS-sorted CD4^+^CD25^+^GFP^+^ DEREG T cells were cultured with wild-type or *p19^−^^/^^−^* BMDC plus soluble α-CD3 in the presence of curdlan. Cells were restimulated on days 5 and 7 with PMA, ionomycin and brefeldin A for 4 h and the expression of Foxp3 and IL-17 on CD4^+^ cell blasts was analyzed by flow cytometry. (B) Total numbers of Foxp3^+^IL-17^+^ T cells and CD4^+^ T cells from cultures as in (A) on day 5. (C) RT-PCR analysis of transcripts encoding ROR-γt, and IL-23R in cultures as in part A and analyzed on day 5. (D) CD4^+^CD25^+^GFP^+^ DEREG T cells were cultured for 5 days with wild-type BMDC plus soluble α-CD3 in the presence of curdlan or IL-23 (10 ng/mL). Cells were restimulated on day 5 with PMA, ionomycin and brefeldin A for 4 h and the expression of Foxp3 and IL-17 on CD4^+^ cell blasts was analyzed by flow cytometry. (E) Numbers of Foxp3^+^IL-17^+^ T cells obtained in cultures as in part D. Data in part B and E are mean+SEM of three independent experiments. Data are representative of two to four independent experiments. N.D., not done.

We have previously shown that curdlan stimulation of DC leads to production of IL-23 but not IL-12 p70 [4]. To address the role of IL-23 in the generation of Foxp3^+^IL-17^+^ T cells, we cultured IL-23-deficient (*p19^−^^/^^−^*) or wild-type BMDC with Foxp3^+^ T cells from DEREG mice in the presence of curdlan. Notably, the frequency and number of Foxp3^+^IL-17^+^ T cells was markedly decreased when *p19^−^^/^^−^* BMDC were used as APC (Fig. 4A and B) while CD4^+^ T-cell expansion was unaffected (Fig. 4B). The appearance of ROR-γt and IL-23R transcripts in curdlan-containing cultures was also IL-23 dependent (Fig. 4C). When curdlan was replaced with IL-23, we obtained similar frequencies of Foxp3^+^IL-17^+^ T cells (Fig. 4D) but T-cell expansion was reduced, resulting in lower numbers of Foxp3^+^IL-17^+^ T cells at the end of the culture (Fig. 4E). These data suggest that IL-23 is necessary but not sufficient for maintaining the phenotype and/or survival of Foxp3^+^IL-17^+^ T cells.

## Discussion

Innate signals play a key role in determining the class of adaptive immune response against infection. In this study, we have shown that innate stimulation *via* the dectin-1 pathway allows DC to instruct CD25^+^Foxp3^+^ T cells to become Foxp3^+^IL-17^+^ T cells. This process is distinct from the differentiation of naïve T cells in curdlan-containing cocultures [4], which generates Th17 cells that do not express Foxp3 (data not shown). Previous reports have demonstrated that Foxp3^+^ T cells can be converted into Th17 cells *in vitro* and *in vivo* under certain conditions [17–20]. In those studies, Foxp3^+^ T cells may pass through a transient stage characterized by the coexpression of Foxp3 and IL-17 before downregulating Foxp3. A distinct hallmark of the cells described in this study is that they maintain or even increase the expression of Foxp3 while acquiring the ability to produce IL-17.

Dectin-1 signaling in DC induces the production of several cytokines, including IL-2, IL-6, IL-10, TNF-α and IL-23 [4]. We have shown that IL-23 is essential to induce but not sufficient to sustain the generation of Foxp3^+^IL-17^+^ T cells. IL-23 might act synergistically with IL-6 and TNF-α, both of which contribute to the generation of Th17 cells [12]. Dectin-1-activated DC also produce IL-2, which could contribute to the generation of Foxp3^+^IL-17^+^ T cells. Indeed, although IL-2 acts as an inhibitor of Th17 differentiation from naïve CD4^+^ T cells [26], it potentiates the expression of Foxp3 in Treg [27]. We propose that the unique balance of cytokines induced in DC by dectin-1 stimulation may be particularly suited to the generation of Foxp3^+^IL-17^+^ T cells, with IL-23, TNF-α and IL-6 favoring IL-17 production and IL-2 sustaining Foxp3 expression. IL-10, which is also produced at high levels by DC stimulated *via* the dectin-1/Syk pathway [4, 28, 29], could potentially help maintain Treg markers and/or Treg activity.

Foxp3^+^IL-17^+^ T cells arise from the Foxp3^+^ROR-γt^+^ Treg subset and not the Foxp3^+^ROR-γt^−^ population, indicating that the Foxp3^+^ROR-γt^+^ population possesses the ability to produce IL-17 under certain conditions of innate immune challenge. In turn, this implies that Foxp3^+^ T cells are not being genetically reprogrammed to become IL-17 producers but that the appropriate cytokine milieu can trigger IL-17 production in cells that already express the genetic machinery required to perform that function.

The fact that conditions of innate stimulation with fungal products favor the development of a novel population of Foxp3^+^IL-17^+^ cells may have important implications. Fungal infections have been linked to IL-17 responses [4] and IL-17-deficient mice are more susceptible to infection with *Candida albicans* [30]. In addition, human memory CD4^+^ T cells specific for *C. albicans* are skewed toward Th17 [3, 31] and STAT3-mediated Th17 deficiency in humans is associated with increased susceptibility to *C. albicans* infection [32]. Whether Foxp3^+^IL-17^+^ T cells participate in anti-fungal immunity or whether they retain regulatory activity that could counteract the detrimental effects of overexuberant Th17 responses [33] remains to be determined. Consistent with the latter possibility, it has previously been reported that DC activated by yeast particles induce T cells with regulatory activity [29]. Notably, small numbers of cells coexpressing Foxp3, ROR-γt and IL-17 have been previously noticed in mouse gut lamina propria [21]. By developing techniques for enriching and isolating Foxp3^+^IL-17^+^ T cells from animals and patients, we hope that multiple questions regarding their role can begin to be addressed.

## Materials and methods

### Mice

C57BL/6 mice were obtained from Charles River. DEREG mice [24] were bred at Technische Universität München or at Cancer Research UK in specific pathogen-free conditions. IL-23 p19-deficient mice [34] were provided by A. MacDonald (Edinburgh, UK) with kind permission from N. Ghilardi, Genentech, and were bred at Cancer Research UK in specific pathogen-free conditions. *Rorc*(*γt*)*-Gfp^TG^* mice [22] were bred at Institut Pasteur. Radiation chimeras using BM from DEREG or *Rorc*(*γt*)*-Gfp^TG^* mice were generated at Cancer Research UK. All animal experiments were performed in accordance with national and institutional guidelines for animal care.

### *In vitro* T-cell assays

CD4^+^CD25^−^CD62L^hi^CD44^lo^ (naïve) and CD4^+^CD25^+^ cells from C57BL/6 mice or CD4^+^GFP^+^CD25^+^ and CD4^+^GFP^−^CD25^−^ cells from DEREG mice or DEREG BM chimeras were purified from spleen and lymph nodes. CD4^+^GFP^+^CD25^+^FR4^+^CD45.1^−^ and CD4^+^GFP^−^CD25^+^FR4^+^ CD45.1^−^ cells from *Rorc(γt)-Gfp^TG^* BM chimeras or CD4^+^CD25^+^FR4^+^ and CD4^+^CD25^−^FR4^−^ cells from C57BL/6 mice were purified from mesenteric lymph node. Cell purification was performed by cell sorting using a MoFlo (Dako Cytomation) or a FACSAria cell sorter (BD Biosciences); 5×10^4^ sorted T cells were cocultured with 2×10^4^ BMDC generated with GM-CSF as described previously [4] and 0.2 μg/mL of soluble anti-CD3ɛ in the presence or absence of 50 μg/mL curdlan (Wako; suspended in PBS at 10 mg/mL), 0.5 μg/mL CpG oligonucleotide 1668 (CpG; Sigma) or 10 ng/mL IL-23 (eBiosciences).

Th17 control consisted of CD4^+^GFP^−^CD25^−^ T cells from DEREG mice or DEREG BM chimeras cocultured with BMDC in the presence of 10 ng/mL of TGF-β (Sigma), 20 ng/mL of IL-6 (RnD systems) and neutralizing antibodies against IFN-γ (2 μg/mL) and IL-4 (2 μg/mL). Treg control consisted of CD4^+^GFP^+^CD25^+^ cells from DEREG mice or DEREG BM chimeras cocultured with BMDC in the presence of 100 IU/mL of rhIL-2 (RnD systems). In some experiments, sorted CD4^+^GFP^+^CD25^+^ T cells were labeled with 2 μM CFSE for 12 min at 37°C before culture.

Cells from cultures were restimulated on day 5 or 7 for 4 h with phorbol 12-myristate 13-acetate (10 ng/mL; Sigma), ionomycin (1 μg/mL; Calbiochem) and brefeldin A (5 μg/mL; Sigma). Intracellular staining for Foxp3 and IL-17 was analyzed by flow cytometry. Alternatively, half of the content of each well were restimulated on day 5 on plate-bound anti-CD3ɛ (5 μg/mL) for 48 h before cytokines in the supernatant were analyzed by sandwich ELISA.

### Flow cytometry

Antibodies specific for CD4 (RM4-5), CD25 (PC61), CD62L (MEL-14), CD44 (IM7) and CD3ɛ (145-2C11) were from BD Pharmingen. Antibodies against Folate receptor 4 (12A5), IL-17 (TC11-18H10.1) and Foxp3 (FJK-16s) were from eBiosciences. For intracellular cytokine staining, cells were stained with anti-CD4, fixed and stained with anti-mouse/rat Foxp3 staining set (eBiosciences) containing fluorochrome-labeled antibodies. Data were acquired on a FACSCalibur (BD Biosciences) and analyzed using FlowJo software (Treestar).

### RNA isolation and real-time RT-PCR

Total RNA was prepared with TRIzol reagent (Invitrogen). cDNA was synthesized from total RNA with random hexamers and Superscript II reverse transcriptase (Invitrogen). Quantitative real-time PCR was carried out using SYBR green incorporation (*Il17f, ROR-*(γ*t*), *Il23r*). For SYBR green reactions, primer sequences were as described previously [2, 5]. Measurements were performed in duplicate wells using the ABI PRISM 7700 sequence detection system (Applied Biosystems). Normalization was performed using 18S rRNA as a reference (primers and probe from Applied Biosystems) and results are shown as relative mRNA quantities.

## Abbreviations:

BMDC: BM-derived DC
DEREG: depletion of regulatory T cell
Th17: Th cells producing IL-17
